# Supplementary material for: Extravasation and fluid collection on computed tomography imaging in patients with colonic diverticular bleeding
Source: PLoS One. 2020 Apr 9;15(4):e0229884. doi: 10.1371/journal.pone.0229884 (PMC7145113; doi:10.1371/journal.pone.0229884)
Supplement: Attachment — Submitted filename: Response to Reviewers.docx [file pone.0229884.s001.docx]

February 3rd, 2020

Dr. Anita Estes

Editor, *PLOS ONE*

Reviewers, *PLOS ONE*

Submission ID: PONE-D-19-28894

Title: Extravasation and fluid collection on computed tomography imaging in patients with colonic diverticular bleeding

Thank you very much for your letter. We appreciate receiving this opportunity to re-submit our manuscript. We have rewritten the manuscript in accordance to the comments. I believe that these changes will alleviate the concerns of the reviewers. We would greatly appreciate if our manuscript could be accepted for publication in *PLOS ONE*.

Sincerely,

Hitomi Takada, MD.

Department of Gastroenterology and Hepatology

Municipal Hospital of Kofu

Yamanashi, Japan

Dear Editor and the reviewers,

Thank you for your kind review of our manuscript.

Considering all the suggestions, the manuscript is revised. We believe that our manuscript is now suitable for the publication in the journal.

1) Please update this statement to indicate whether all data were fully anonymized before the authors accessed them and whether the IRB or ethics committee waived the requirement for informed consent.

Thank you for your comment. The institutional review board of Municipal Hospital of Kofu approved the study (ethics committee for clinical studies of Municipal Hospital of Kofu : Rinshoukenkyu-Rinrishinsa-Iinkai (in Japanese), approval number 31-24), the ethics committee waived the requirement for informed consent. All data were fully anonymized before we accessed them. This is now modified in the methods session. (page3, lines 90-94)

2) You note that your data are available within the Supporting Information files, but no such files have been included with your submission. At this time we ask that you please upload your minimal data set as a Supporting Information file, or to a public repository such as Figshare or Dryad.

Thank you for your comment. All data are within the manuscript, and we have no additional supporting files.
